# Reducing the Mobility of SARS-CoV-2 Variants to Safeguard Containments

**DOI:** 10.1007/s10272-021-0987-4

**Published:** 2021-08-05

**Authors:** Martin Hellwig, Viola Priesemann, Guntram B. Wolff

**Affiliations:** 1 Max Planck Institute for Research on Collective Goods, Kurt-Schumacher-Str. 10, 53113 Bonn, Germany; 2 Max Planck Institute for Dynamics and Self-Organization, Am Faßberg 17, 37077 Göttingen, Germany; 3 Bruegel, Rue de la Charité 33, 1210 Brussels, Belgium

## Abstract

Escape variants can cause new waves of COVID-19 outbreaks and put vaccination strategies at risk. To prevent or delay the global spread of these waves, virus mobility needs to be minimised through screening and testing strategies, which should also cover vaccinated people. The costs of these strategies are minimal compared to the costs to health, society and the economy from another wave.

When the coronavirus pandemic started in 2019/2020, a number of countries reacted early, closing down public life and reducing private contacts before contagion fully took off. Countries that failed to do this saw large spikes in cases, stretching or overwhelming their medical capacities. Likewise, countries that ignored warning signals of a second wave were hit hard in autumn 2020. A third wave, caused by the more contagious B.1.1.7 SARS-CoV-2 variant, has unfolded. This variant was first recognised in Kent, United Kingdom, from where it spread quickly across the UK and beyond. It spread to countries with more travel to the UK earlier than others. Germany was affected relatively late, while Portugal and Ireland were affected early because of more intensive travel links.

Another variant, P1, is also spreading rapidly in Brazil and countries with strong travel connections to Brazil, such as Chile.

**Guntram B. Wolff**, Bruegel, Brussels, Belgium.

People travelling play a central role in spreading new variants of SARS-CoV-2, with devastating consequences. Stopping a new variant from entering from abroad, or at least slowing it down, would facilitate containment and limit the human, social and economic costs. The experience with B.1.1.7 shows how slower entry of a variant to a country delays the deterioration of the health situation and the introduction of strict and costly lockdowns.

Public policy in advanced economies is focusing on vaccination in the hope this will bring down the number of severe cases and deaths while allowing restrictions to be lifted (Dagan et al., 2021). By the end of 2021, large parts of the populations of Israel, Chile, the US, the UK and the EU will have received the vaccine and will be largely immune to the wild strain and some variants of SARS-CoV-2.

However, additional waves of contagion must be expected, caused in particular by escape variants against which current vaccines are less effective (McCormick et al., 2021). Even if vaccination continues as currently foreseen, the virus will not be fully eliminated. With incomplete uptake of vaccines, waning immunity and imperfect transmission prevention, it will persist in certain sub-populations (Phillips, 2021). Moreover, in many countries, vaccination is proceeding slowly if at all. The persistent prevalence of the virus in various sub-populations and various places provides a breeding ground for mutations. With advances in vaccination and immunisation, variants that escape the immune response will have an evolutionary advantage. The emergence of escape variants has been documented in several regions, and a similar evolution has also been observed in vitro (Andreano et al., 2020).

With an escape variant, contagion persists in the vaccinated population itself and new escape-variant waves could spread very quickly if a large part of the population has been vaccinated and moves about without restraint. New restrictions, up to complete lockdowns, might therefore become inevitable, until a new vaccine is developed and administered, and immunisation against the new variant is successful.

Any strategy that relies on vaccination only will therefore be insufficient. While everything should be done to speed up the supply of vaccines and their roll-out, a strategy that recognises and slows down the emergence of new variants and limits their spread is needed. Such strategies must encompass the part of the population that has acquired immunity against the wild strain, through vaccinations or through infections.

Preparing for this potential danger requires a strategy with three central goals: (1) minimising the rate at which escape variants develop, (2) detecting them early and (3) minimising the mobility of the virus. While all three goals are in principle technically feasible, they run against basic human and social needs, in particular in open societies. Measures to meet the goals must therefore be designed in a way that renders them acceptable. These measures will also be substantially cheaper economically than sustained contagion waves.

### Minimising the rate at which escape variants develop: Minimising SARS-CoV-2 incidence

Low incidence is the best protection against the breeding of new variants. Roughly, the probability that an escape variant emerges is proportional to the number of infected people, and the expected time until a variant emerges is inversely proportional to the number of infected people. Reducing the number of infected people delays the expected emergence of a new escape variant. Low case numbers also have clear advantages for public health, society and the economy (Priesemann et al., 2021; Oliu-Barton et al., 2021). Moreover, at low case numbers, contact tracing contributes efficiently to containment, allowing the health authority to concentrate on the remaining infection chains (Contreras et al., 2021). Lastly, local outbreaks of new variants are detected early and not hidden within a generally high incidence.

However, even with low case numbers, escape variants can emerge. Once this has happened, a new variant's effective reproduction rates in the immunised and non-immunised parts of the population depend not only on the characteristic basic reproduction numbers $R_0$ and $R_0'$, but also on the contact and hygiene behaviour in the two parts of the population. Assuming that the immunised population is less careful about protecting itself, a potent variant could spread very quickly, because the effective reproduction rate is very high. If such an outbreak is not detected and fought early, development of a new vaccine will be too late, after the wave has taken its toll.

### Early detection of escape variants: Screening and surveillance

The early, local detection of virus variants is important to slow their global spread. Regular screening of a representative sample of the population, as established in the UK for example, provides a better basis for informed scientific evaluation of the pandemic than only the current, more symptom-based testing. Screening would contribute to an early warning system for the emergence of new outbreaks and new variants, with data shared rapidly across the globe (Cyranoski, 2021).

For COVID-19, testing should also include immune and vaccinated people, because infection of vaccinated individuals by escape variants could be particularly fast because of their higher numbers of contacts. Testing reports should list the immunisation status of those tested (e.g. date and type of vaccine, earlier infection). This facilitates detection of outbreaks among the vaccinated population – a key indicator for an escape variant.

To reach a representative part of the population, testing should be organised at schools and workplaces where it would be embedded in an organised setting and can be implemented as part of a daily or weekly routine. In addition, testing at schools and workplaces would target population groups that have regular contacts outside the household. Incentives for regular testing matter for compliance. For employers, the desire to avoid interruptions of production activities caused by employee illness, quarantine or long COVID provides an incentive for testing.

Governments need to take legal steps to facilitate workplace testing and perhaps even impose it. In schools, teachers have an interest in testing to avoid self-infection. Complete testing of entire groups with single PCR test, where all samples are jointly and anonymously evaluated, could forestall concerns about privacy, ensure higher accuracy than individual self-tests, and reduce costs. Overall, easily accessible testing, together with systematic virus genome surveillance, is a core activity to detect new variants early.

### Reducing the virus spread: Testing and quarantine

If an escape variant emerges and spreads, it makes a huge difference whether, at the time it is recognised, the number of infected individuals is 10 or 1000. Assuming an R of 1.4 and a serial interval of four days, then starting from 10, rather than 1000 individuals, buys almost eight weeks to implement mitigation strategies and adapt vaccinations.

The difference between 10 and 1000 initial carriers can be achieved by installing a testing strategy that prevents 99% of virus carriers from entering a region. Systematic testing of travellers would be a central part of this. Such testing must encompass immunised and non-immunised people, as both groups might transmit the virus.

A strategy to prevent a virus's rapid spread via human mobility should in principle apply to within-jurisdiction travel as well as cross-border travel. Some might object that we do not ordinarily test for as yet unknown diseases. However, escape variants are different from as yet unknown viruses. They are sufficiently similar to the variants we know to be detectable through testing. That opportunity should be used.

## Is this strategy feasible?

None of our suggestions involves a major economic cost. Relative to the cost of travel, a standard test would increase the cost of a flight by perhaps around €40 – and would require the time it takes the individual to get tested. The costs are thus not zero but are small relative to the costs of further waves of contagion: the economic costs of lockdowns and similar restrictions, let alone an uncontrolled pandemic, are many times the costs of the measures we propose (Cutler and Summers, 2020).

The real challenge is societal. Testing requirements run counter to a sense of individual rights deeply rooted in Western societies. The dismantling of borders and border controls are considered major advancements.

However, frictionless travel comes at a cost and involves risks of contagion. Once contagion takes off, the implications for individual freedom are much more serious than the restrictions from testing requirements attached to travel. Certification requirements for cross-border movements of people do not prevent travel. For transport of food and animals, such requirements are considered normal.

The political challenge is to communicate to the public that testing for viruses does not end mobility but is a way of keeping societies open. The proposed testing strategy buys limited time. To use this time sensibly, public health measures are necessary to slow down the spread and flatten the curve, while increasing research and production capacity for effective vaccines (for example with market design; see Castillo et al., 2021).

## Conclusion

Overall, it is of critical importance to prevent future waves of escape variants hitting unprepared societies in the coming months. The basic requirement is rapid progress on vaccination and low case numbers across the world (Priesemann et al., 2021). This should be complemented by a rigorous testing strategy that stops viruses from spreading while keeping borders open to human travel. In this way, repeated lockdowns and their large health, economic, social and personal costs, which dwarf any costs related to testing, can be avoided. Given the risk of future waves, Western societies in particular must learn that unprotected travel can have much larger social costs than hitherto accepted and must hence start rigorous prevention against the spread of SARS-CoV-2 variants.
